# Commentary: DNA Damage Promotes Epithelial Hyperplasia and Fate Mis-specification via Fibroblast Inflammasome Activation

**DOI:** 10.29245/2767-5092/2022/2.1154

**Published:** 2022-06-28

**Authors:** David Solano, Kush R. Patel, Adelaida B. Perez, Lindsey Seldin

**Affiliations:** 1 Department of Cell Biology, Emory University School of Medicine, Atlanta, GA, USA; 2 Department of Dermatology, Emory University School of Medicine, Atlanta, GA, USA; 3 Winship Cancer Institute, Emory University School of Medicine, Atlanta, GA, USA; 4 Atlanta Veterans Affairs Medical Center, Decatur, GA, USA

**Keywords:** Inflammasome, Fibroblast, Microenvironment, Stem cell, Epithelia, Homeostasis, Hyperplasia, Wound healing, DNA damage, Quiescence, IL-1β, Skin cancer, Chemotherapy

## Introduction

Epithelial tissues, while diverse in form, share a critical barrier function that must be properly established throughout development, maintained during homeostasis, and restored following injury. The barrier function of the epidermis, the largest epithelial organ, is essential for animal survival, serving as the body’s outermost protective layer that prevents pathogen entry while promoting fluid retention. Disruptions to epidermal homeostasis require the rapid replacement of lost or damaged cells to efficiently restore barrier integrity. This regenerative capacity relies on quiescent stem cell populations that are primed to proliferate and able to generate the diverse cell types required for tissue function. Nevertheless, stem cell proliferation and plasticity must be stringently controlled, as dysregulation of these behaviors could have tumorigenic consequences^1, 2^.

**Figure 1.**
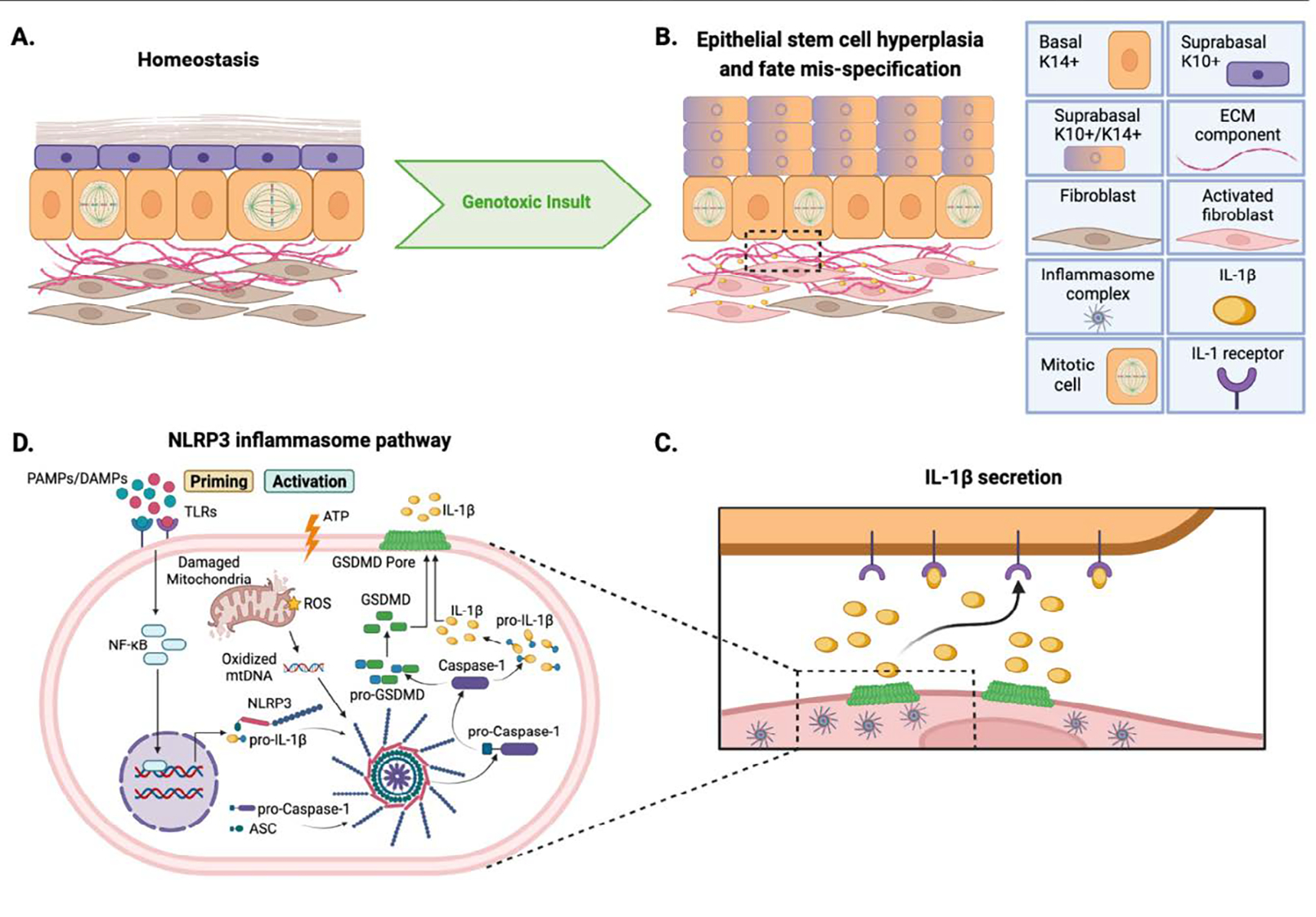
Impact of Genotoxic Damage on Epidermal Stem Cell Behavior. A) Adult mouse epidermis in homeostasis. B) Epidermis following genotoxic treatment, highlighting basal cell hyperplasia, suprabasal cell fate mis-specification, and fibroblast inflammasome activation as reported in Seldin and Macara 2020. C) Zoom in of boxed region in (B) showing hypothetical binding of fibroblast-secreted IL-1β to IL-1 receptors on epithelial basal cells. D) NLRP3 inflammasome pathway schematic, highlighting both intrinsic (mitochondrial damage-driven) and extrinsic (Toll-like receptor-driven) mechanisms of pathway activation. IL, interleukin; PAMP, pathogen-associated molecular pattern; DAMP, damage-associated molecular pattern; TLR, toll-like receptor; GSDMD, Gasdermin D; mtDNA, mitochondrial DNA; NLRP3, NOD-, LRR-and pyrin domain-containing protein 3. Created with BioRender and Adobe Illustrator.

The epidermal epithelium overlies a basement membrane that separates it from the underlying dermis (Fig. 1A). The dermal microenvironment contains a repertoire of diverse cell types, including fibroblasts and immune cells, that promote wound-healing by secreting growth factors and inflammatory cytokines to stimulate epithelial cell expansion, re-epithelization, and basement membrane remodeling required for efficient wound closure^3^. Notably, wound-healing and inflammatory mechanisms critical for re-establishing tissue homeostasis are often hijacked by cancer cells to drive tumor expansion^4, 5^. Recent research has made important progress in dissecting how epithelial/dermal crosstalk modulates epidermal stem cell regulation in diverse contexts, including development, wound-healing, aging, and disease^6–11^. A thorough mechanistic understanding of this crosstalk is critical for both optimizing regenerative treatments for wounds, burns and/or genetic skin conditions^12^, as well as developing targeted therapies for cutaneous cancers and other diseases.

**Figure 2.**
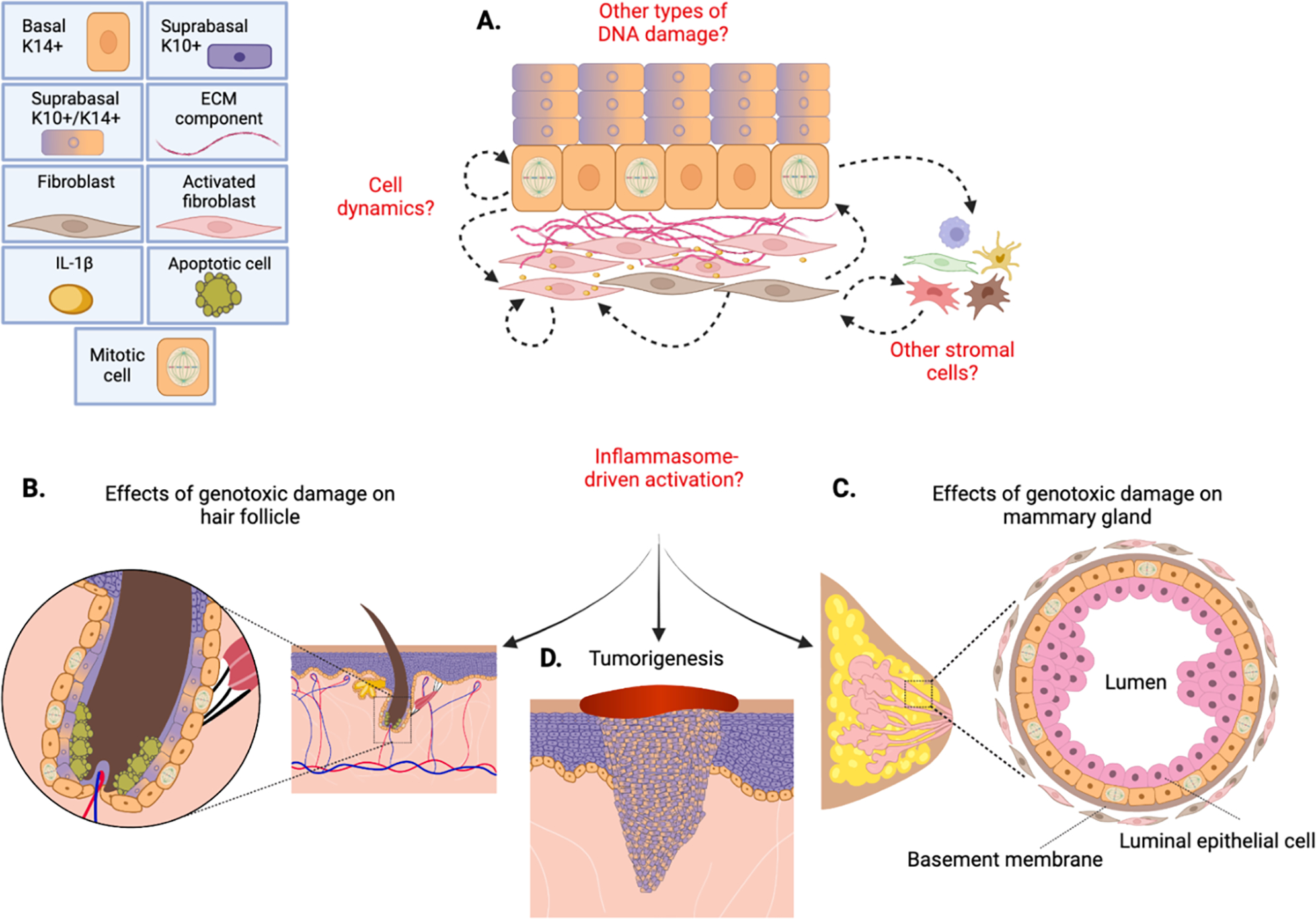
Potential Mechanisms of Genotoxic-Driven Epithelial Behaviors. A) Epidermis following genotoxic treatment. Dotted arrows indicate potential cell-cell interactions, signaling and dynamics that may facilitate the epidermal responses to DNA damage. B-D) Genotoxic damage-associated epithelial behaviors in the hair follicle (B) and mammary gland (C) as reported in Seldin and Macara 2020, as well as tumorigenesis (D), may involve inflammasome signaling. Created with BioRender and Adobe Illustrator.

Recently published findings by Seldin and Macara^13^ elucidated a novel mechanism of epithelial/dermal crosstalk whereby DNA damage triggers innate immune signaling in fibroblasts within adult mouse backskin; this, in turn, stimulates proliferation and alters fate determination in epidermal stem cells (Fig. 1). By applying transgenic, lineage-tracing, fibroblast transplantation and RNA sequencing approaches, this study revealed that fibroblast-specific interleukin-1β (IL-1β), by way of NOD-, LRR- and pyrin domain-containing protein 3 (NLRP3) inflammasome pathway activation, is both necessary and sufficient for the epidermal response to DNA damage (Fig. 1). In this commentary, we will review the novel conclusions of this work and their limitations, discuss relevant future directions (Fig. 2), as well as speculate on potential clinical implications.

## The Counter-Intuitive Effect of DNA Damage on Stem Cell Behavior

The main findings in Seldin and Macara 2020 are paradoxical. The DNA crosslinking agents applied in this study, which include cisplatin and mitomycin, have proven effective as chemotherapies to treat epithelial cancers due to the irreparable DNA damage they cause in rapidly dividing cancer cells^14^. This damage prevents the proper function of DNA and RNA polymerases, ultimately resulting in the activation of p53 and other pro-apoptotic signaling pathways to facilitate tumor regression^15^. Surprisingly, this study reported that these crosslinking agents do not prompt apoptosis in wild-type adult mouse skin, but instead promote quiescent epithelial cells to enter the cell cycle and become hyperplastic. The contrary effects of these agents based on cell cycle status were demonstrated by experiments in the hair follicle, where proliferative matrix cells at the follicle base were robustly ablated following treatment, while neighboring non-dividing outer root sheath epithelia became hyper-proliferative (Fig. 2B). Furthermore, when cultured ex vivo, proliferating epithelial stem cells died upon cisplatin exposure.

The destructive effect of genotoxic agents on diverse proliferative cell populations, both normal and tumorigenic, likely underlies the adverse side effects of chemotherapy experienced by cancer patients, such as hair loss^16^. This study’s findings underscore the necessity of refining treatment approaches so that only cancer cells are targeted for destruction. Furthermore, these discoveries may have important clinical implications for recurrence and/or chemotherapeutic resistance in epithelial cancers; dormant cells adjacent to tumors might undergo activation and expansion whilst rapidly dividing cancer cells are eliminated^17^. It is important to note that the data in Seldin and Macara 2020 are limited to localized, short-term drug treatments in wild-type tissue. Future studies applying long-term, repeat and/or systemic treatment protocols, cancer mouse models, as well as human patient data may provide important mechanistic insights for improving chemotherapeutic efficacy.

## NLRP3 Inflammasome Activation in Dermal Fibroblasts

Another surprising finding from Seldin and Macara 2020 is that dermal fibroblasts, and not immune cells, exhibit NLRP3 inflammasome activation following DNA damage (Fig. 1). In fact, an intact immune system was not required for the DNA damage response; mouse skin deficient in both innate and adaptive immunity demonstrated similar epithelial expansion and stem cell fate changes compared to wild-type tissue. Prior to this study, inflammasome signaling had been largely attributed to macrophages and other immune cells^18^, although epidermal epithelia were previously shown to express Aim2^19^, NLRP1^20^, and NLRP3^21^ inflammasomes. Nevertheless, inflammasome activity had not been reported in normal adult dermal fibroblasts.

The NLRP3 inflammasome is a multiprotein signaling module that can be activated via extrinsic and/or intrinsic mechanisms; extracellular damage signals can bind cell surface Toll-like receptors to initiate NFκB signaling, and/or intracellular reactive oxygen species (ROS) can cause mitochondrial damage resulting in the release of oxidized mitochondrial DNA into the cytoplasm (Fig. 1D). These damage stimuli drive inflammasome oligomerization, caspase 1 activation, and subsequent IL-1β and IL-18 cleavage and secretion through Gasdermin D pores^22^ (Fig. 1D). In Seldin and Macara 2020, dermal immunostaining following DNA damage revealed fibroblast-specific NLRP3 inflammasome activation, a finding corroborated by RNA sequencing. Furthermore, experiments using IL-1β-specific blocking antibodies or purified IL-1β injections into the dermis confirmed, respectively, that this potent cytokine is necessary and sufficient for the DNA damage response. Notably, although inflammasome activation has been associated with pyroptosis^23^, the successful transplantation and tracking of fibroblasts following DNA damage indicate that IL-1β secretion is not always concomitant with cell death. Taken together, these findings suggest that the skin DNA damage response is primarily driven by activation of a noncanonical fibroblast-specific NLRP3 inflammasome. Nevertheless, further investigation is required to clarify whether this mechanism is a generalizable response to diverse forms of DNA damage, if the fibroblast inflammasome is activated via extrinsic and/or intrinsic means, if fibroblast IL-1β signals directly by binding epithelial cell receptors (Fig. 1C), and whether additional stromal cell populations help initiate and/or propagate this response (Fig. 2A).

## Conserved Epithelial DNA Damage Response

Intriguingly, similar stem cell phenotypes were observed in both hair follicle and mammary gland epithelia, suggesting a conserved response to DNA damage (Fig 2B–C). In the mammary gland, which consists of an outer layer of unipotent myoepithelial basal cells and an inner layer of unipotent luminal cells^24^, quiescent basal cells became hyperplastic and exhibited enhanced plasticity upon DNA damage by generating luminal cell progeny (Fig 2C). This caused tissue disorganization via luminal filling, reminiscent of cell behaviors during an early stage of breast cancer called ductal carcinoma in situ. It remains unclear, however, whether fibroblasts and/or inflammasome signaling underlie the aberrant mammary cell behaviors observed in this study, and whether the mechanism is generalizable to other reported mammary damage responses^25^. Since enhanced breast cancer cell plasticity underlies intratumor heterogeneity^26^, a major challenge to achieving therapeutic efficacy, follow-up studies that further dissect regulators of mammary cell plasticity are crucial^27^.

## Disease Implications

The discovery that DNA damage-driven cytokine signaling from fibroblasts can dramatically impact homeostasis in diverse epithelia begs the question of whether cancer-associated fibroblasts (CAFs) may utilize a similar mechanism to drive epithelial tumorigenesis (Fig. 2D). Importantly, damage response mechanisms such as inflammation have been intimately linked to tumorigenesis^28^, IL-1 has been associated with cancer progression^29^, and DNA damage is the primary driver of cutaneous skin carcinomas. While skin CAFs have been understudied, pancreatic CAFs were recently reported to assume an inflammatory nature (“iCAFs”) in response to IL-1 signaling^30, 31^ and breast cancer CAFs can exhibit inflammasome activation^32^. These studies imply that IL-1β signaling from dermal fibroblasts might also contribute to skin cancer development and/or progression, as hinted by previous work on squamous cell carcinoma^11^. Furthermore, inflammasome activation may also be involved in skin inflammatory disorders such as psoriasis and atopic dermatitis, which produce a similar tissue expansion phenotype as that observed in Seldin and Macara 2020 but are assumed to be driven by immune cell signaling. While innovative new therapies targeting the inflammasome, including inflammasome-specific nanobodies^33^, are being developed to treat a broad range of autoinflammatory diseases, their cancer therapeutic potential remains undetermined^34^.

## Conclusion

Seldin and Macara 2020 unveiled important implications for fibroblast inflammasome signaling in the etiology of epithelial disease. Nevertheless, additional work incorporating disease mouse models and patient-derived xenografts would help clarify whether this study’s findings are clinically relevant. Furthermore, several mechanistic gaps remain regarding how the inflammasome modulates epithelial stem cell proliferation, plasticity, fate decisions and quiescence. Whether inflammasome activity can serve as a biomarker of disease and/or be harnessed for regenerative medicine are intriguing topics for future investigation.
